# Pleuropulmonary Blastoma (PPB) in Child with *DICER1* Mutation: The First Case Report in the State of Qatar

**DOI:** 10.1155/2021/1983864

**Published:** 2021-10-29

**Authors:** Sara G. Hamad, Amal Al-Naimi, Mutasim Abu-Hasan

**Affiliations:** Pediatric Pulmonology, Sidra Medicine, Doha, Qatar

## Abstract

Pleuropulmonary blastoma (PPB) is a rare intrathoracic malignancy, which arises from the lung parenchyma and/or pleura. PPB has strong genetic association with mutations in *DICER1* gene. Despite being rare, PPB is the most common lung tumor in children below 6 years of age. International registry of the disease has a total of 350 cases worldwide. We report the first case of PPB in the state of Qatar, which presented as a large cystic lung lesion. The patient was first thought to have benign congenital pulmonary airway malformation (CPAM) based on chest X-ray findings. The diagnosis of PPB was suspected based on chest CT scan findings and was confirmed after surgical resection of the cystic mass. The case highlights the need to consider PPB in the differential diagnosis of cystic lung lesions in children and the need for further radiological imaging (i.e., CT scan), genetic testing, and/or excisional biopsy to confirm the diagnosis.

## 1. Introduction

Congenital cystic lung lesions are a group of lung diseases which represent variable pathology ranging from benign congenital malformation to neoplasm. These lesions can present with an overlapping clinical and radiological features. Based on the type of the lesion, treatment approach can range from observation to surgical resection. Several classifications have been proposed based on the radiological and pathological features [1]. Pleuropulmonary blastoma (PPB) is an extremely rare and potentially serious subtype of neoplastic cystic lung lesions, which can be misdiagnosed as a benign congenial cystic lesion. Therefore, early diagnosis is essential.

PPB has strong genetic association with mutations in *DICER1* gene. The identification of such mutation in patients with cystic lung lesion can help with diagnosis and guide management. We report the clinical presentation and outcome of the first case of PPB in the state of Qatar with identified DICER1 gene.

## 2. Case Report

**Figure 1:**
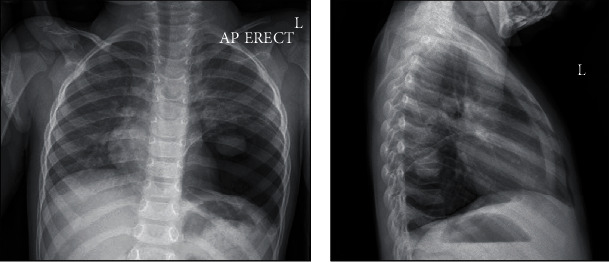
Anteroposterior and lateral chest X-ray showing cystic lesion at left lower zone.

The patient is a 35-month-old boy, previously healthy who presented to emergency department with history of low-grade fever, rhinorrhea, and abdominal pain. There was no history of recurrent chest infections, bone pain, weight loss, or fatigue. Physical examination was significant for decreased breath sounds over posterior aspect of left lower chest zone. Chest X-ray showed large cystic lesion in left lower lobe (Figure 1). No previous chest X-rays were obtained for comparison.

**Figure 2:**
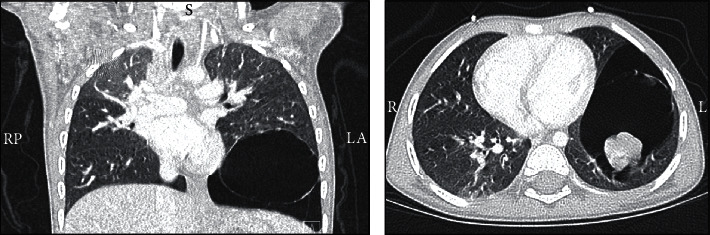
Multiseptated large cystic lesion within the left lower lobe with multiple solid nodules.

Patient was referred to pediatric pulmonology clinic with a preliminary diagnosis of congenital pulmonary airway malformation (CPAM). Therefore, chest computed tomography (CT) was performed and showed multiseptated large cystic lesion within the left lower lobe with multiple solid nodules. Enlarged lymph nodes were also noted in the left paratracheal region; the largest lymph node measured 10 mm (Figure 2). The presence of solid nodules and the enlarged lymph nodes raised the possibility of pleuropulmonary blastoma.

**Figure 3:**
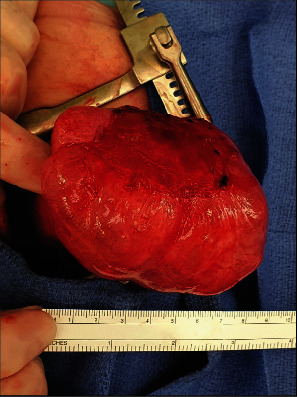
Intraoperative gross section of lesion.

Resection of the lesion via left lower lobectomy was done through left thoracotomy. A large cyst measuring 8 × 8 × 10 cm was seen within the left lower lobe with adherence to left hemidiaphragm, but no evidence of invasion of the chest wall or the diaphragm (Figure 3).

**Figure 4:**
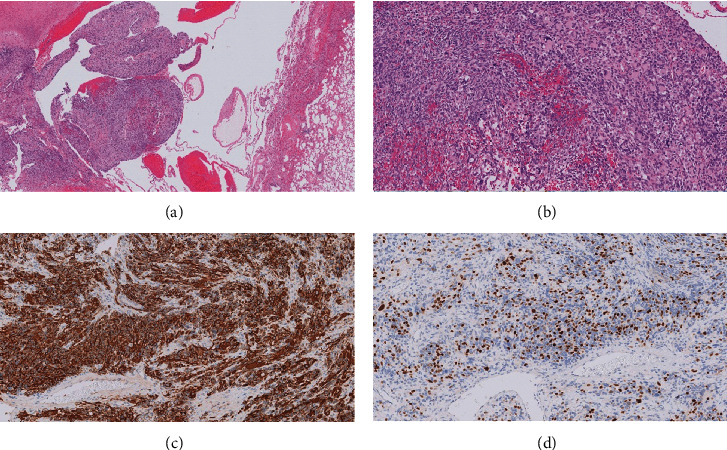
(a) Solid and cystic components of the tumor (low definition with hematoxylin and eosin stain). (b) Atypical rhabdomyoblasts in solid component (high definition with hematoxylin and eosin stain). (c) Diffuse strong cytoplasmic expression with desmin stain. (d) Myogenin nuclear expression in rhabdomyoblasts.

Histopathological examination of the mass revealed multiloculated cystic and solid components containing atypical rhabdomyoblasts with diffuse cytoplasmic expression of desmin and patchy nuclear expression of myogenin (Figure 4). The pathology confirmed the diagnosis of type II PPB. Excisional margins were safe. One of the excised lymph nodes showed metastatic cells. Whole body MRI (including brain MRI) and whole body PET CT scan revealed no evidence of distant metastatic lesions.

Patient had uneventful postoperative course. Chemotherapy was commenced according to the recommendation of the International Pleuropulmonary Blastoma Registry (IPPBR), which consists of four agents (ifosfamide, vincristine, actinomycin-D, and doxorubicin) over a period of 36 weeks.

The patient completed a year of the disease-free period as evidenced by normal chest CT, normal brain MRI, and normal ultrasound of abdomen and thyroid.

Genetic testing showed that the patient has heterozygous pathogenic mutation in *DICER1* gene. On the other hand, the patient's father and 2-year-old sibling were also tested positive for the same mutation. However, both were completely asymptomatic and had no evidence of lung cysts or masses by chest X-ray screen.

## 3. Discussion

Pleuropulmonary blastoma (PPB) is a rare intrathoracic malignancy arising from the lung parenchyma and/or pleura. It is considered the most common primary intrathoracic malignancy of childhood. PPB was first described in 1988 [2] as a dysembryonic neoplasm of pleuropulmonary mesenchyme [3]. PPB is usually diagnosed in children less than 6 years of age. Most patients are asymptomatic, and the lesions are identified incidentally by chest X-rays obtained for different reasons. Delay in diagnosis, therefore, may occur until disease progresses to advanced stages, causing compressive symptoms, hemodynamic instability, and metastatic lesions [4].

PPB is classified into three subtypes: type I consists only of cystic lesions; type II consists of mixed cystic and solid components, whereas type III consists of only solid tumors. These subtypes represent a progression process based on reported cases of type I progression into type II and type III [5, 6]. The presence of cysts in type I and II PPB may lead to its misdiagnosis as congenital airway malformation (CPAM) or postinfectious pneumatocele. Follow-up chest X-rays, chest CT scan, and surgical excision are important in reaching the correct diagnosis.

Mutation in the DICER1 gene was described in majority of PPB cases. According to the largest International Pleuropulmonary Blastoma Registry (IPPBR), which reported 350 cases, 66% of the 97 tested patients had a heterozygous germline *DICER1* mutation [7]. The *DICER1* mutation status did not affect outcome or prognosis [7]. PPB is considered the most common manifestation of DICER1 mutation tumor predisposition syndrome in children [7]. *DICER1* mutation predisposes to other tumors, such as Sertoli-Leydig cell tumors, ciliary body medulloepithelioma, Wilms tumor, and carcinoma of the thyroid gland [8, 9].

To our knowledge, only five cases of PPB were reported from the Middle East [10–13],, and only one of these cases had DICER-1 gene mutation [14]. Our case is the first reported case of PPB with DICER1 mutation in the state of Qatar. It is worth noting that even though the father and younger sibling of our patient have the same mutation, neither of them has developed mutation-related tumors so far. The risk of life-long tumor formation in individuals with DICER1 syndrome is only moderately increased compared with tumor risk in the general population. However, close follow-up and frequent screening for tumor development in asymptomatic subjects with identified DICER1 gene have been recommended [15].

Many prognostic factors affecting the outcome of PPB were investigated. The subtype of the PPB and the presence of metastases at diagnosis were the only statistically significant unfavorable prognostic factors [9, 16, 17]. There are no reports of metastases in patients with type I-PPB. Therefore, metastatic workup is not required in this subtype. On the other hand, type II and type III PPB have metastatic potential to the brain, bone, and rarely the liver. Therefore, chest computed tomography, brain MRI, abdominal ultrasound, and bone scan are required [18, 19].

The mainstay of PPB management is complete surgical resection of the tumor with or without chemotherapy depending on the subtype [8, 18]. The International Pleuropulmonary Blastoma Registry has reported the overall survival rates for type I-PPB, type II-PPB, and type III-PPB to be 91%, 71%, and 53%, respectively [9].

## 4. Conclusion

PPB is a rare and potentially aggressive, intrathoracic malignancy which can be insidious. Early radiological and histological testing is imperative for early diagnosis and differentiation from benign pulmonary cystic lesions, which would highly influence outcomes and survival.
